# pGluAβ increases accumulation of Aβ in vivo and exacerbates its toxicity

**DOI:** 10.1186/s40478-016-0380-x

**Published:** 2016-10-07

**Authors:** Oyinkan Sofola-Adesakin, Mobina Khericha, Inge Snoeren, Leo Tsuda, Linda Partridge

**Affiliations:** 1 Institute of Healthy Ageing, and G.E.E., University College London, Darwin Building, Gower Street, London, WC1E 6BT UK; 2 Max Planck Institute for Biology of Ageing, Joseph-Stelzmann-Straße 9B, 50931 Cologne, Germany; 3 Center for Development of Advanced Medicine for Dementia (CAMD), National Center for Geriatrics and Gerontology (NCGG), Obu, Aichi Japan

**Keywords:** Neurodegeneration, Alzheimer’s disease, pyroglutamate Abeta, *Drosophila*

## Abstract

Several species of β-amyloid peptides (Aβ) exist as a result of differential cleavage from amyloid precursor protein (APP) to yield various C-terminal Aβ peptides. Several N-terminal modified Aβ peptides have also been identified in Alzheimer’s disease (AD) brains, the most common of which is pyroglutamate-modified Aβ (Aβ_pE3-42_). Aβ_pE3-42_ peptide has an increased propensity to aggregate, appears to accumulate in the brain before the appearance of clinical symptoms of AD, and precedes Aβ_1-42_ deposition. Moreover, in vitro studies have shown that Aβ_pE3-42_ can act as a seed for full length Aβ_1-42._ In this study, we characterized the *Drosophila* model of Aβ_pE3-42_ toxicity by expressing the peptide in specific sets of neurons using the GAL4-UAS system, and measuring different phenotypic outcomes. We found that Aβ_pE3-42_ peptide had an increased propensity to aggregate. Expression of Aβ_pE3-42_ in the neurons of adult flies led to behavioural dysfunction and shortened lifespan. Expression of Aβ_pE3-42_ constitutively in the eyes led to disorganised ommatidia, and activation of the c-Jun N-terminal kinase (JNK) signaling pathway. The eye disruption was almost completely rescued by co-expressing a candidate Aβ degrading enzyme, neprilysin2. Furthermore, we found that neprilysin2 was capable of degrading Aβ_pE3-42._ Also, we tested the seeding hypothesis for Aβ_pE3-42_ in vivo, and measured its effect on Aβ_1-42_ levels. We found that Aβ_1-42_ levels were significantly increased when Aβ_1-42_ and Aβ_pE3-42_ peptides were co-expressed. Furthermore, we found that Aβ_pE3-42_ enhanced Aβ_1-42_ toxicity in vivo. Our findings implicate Aβ_pE3-42_ as an important source of toxicity in AD, and suggest that its specific degradation could be therapeutic.

**Electronic supplementary material:**

The online version of this article (doi:10.1186/s40478-016-0380-x) contains supplementary material, which is available to authorized users.

## Introduction

Alzheimer’s Disease (AD) is a neurodegenerative disorder characterized by amyloid beta (Aβ) deposits and neurofibrillary hyperphosphorylated tau tangles [1]. The amyloid cascade, which has undergone some revision in recent years, is the leading hypothesis for the pathology associated with AD, and states that amyloidogenic Aβ is the trigger of the pathogenic process leading to neuronal cell death [2, 3]. Aβ induces several stressors, which could lead to neuronal cell death [4]. The c-Jun N-terminal kinase/stress-activated protein kinase (JNK/SAPK) signaling pathway which influences cell death is activated in response to many forms of stress, such as oxidative stress and endoplasmic reticulum (ER) stress [5, 6]. In particular, Aβ is able to activate the JNK/SAPK pathway, and increased phosphorylation of JNK/SAPK has been observed in post-mortem AD brain tissue in comparison to control cases [5, 7].

Aβ peptides form as a cleavage product from the amyloid precursor protein (APP) [8]. Several species of Aβ peptides exist, as a result of differential cleavage from APP to yield various C-terminal Aβ peptides. Aβ40 and Aβ42 are the most abundant, with Aβ42 being the more toxic form [9]. More recently, Aβ43 has also been identified as a pathogenic species [10, 11]. There are also several N-terminal truncated/modified Aβ peptides that have been identified in AD brains, the most common of which is pyroglutamate-modified Aβ [12–14].

Pyroglutamic (pGlu) acid is generated from N-terminal glutamine during pro-hormone maturation in the secretory pathway; the enzyme glutaminyl cyclase (QC) is directed to the secretory pathway and catalyses the conversion from N-terminal glutamic to pGlu acid [15, 16]. Interestingly, Aβ undergoes this post-translational modification at its amino terminus, also catalyzed by QC [17], which is up-regulated in the cortex of patients with AD [18]. Of particular interest is the highly abundant Aβ_pE3-42_, which is generated by cleavage of the first 2 amino acids of the Aβ peptide, followed by pGlu-modification of glutamate in the third amino acid position, which is thought to stabilize it and/or promote its aggregation propensity [14].

Interestingly, water soluble Aβ, which appear before plaques is made up predominantly of Aβ_pE3-42_ [19], and indeed Aβ_pE3-42_ accumulate early on in the brain before the appearance of clinical symptoms and Aβ_1-42_ deposition [20–23]. Moreover, in vitro studies have shown that Aβ_pE3-42_ has an increased propensity to aggregate in comparison to Aβ_1-42_ and can act as a seed/primer for full length Aβ_1-42_ [14, 24, 25]_._ Aβ_pE3-42_ behaves in a prion-like manner, whereby only small quantities of Aβ_pE3-42_ are able to increase the amount of metastable low-n Aβ_1-42_ oligomers in vitro [25]. These attributes have created a lot of interest in Aβ_pE3-42_ peptides, and several groups have suggested that they are important in initiating the pathological cascade of AD [14, 20].

Interestingly, Aβ_pE3-42_ plaque load has been observed in brain autopsies of familial, sporadic cases and controls although, importantly, oligomeric Aβ_pE3-42_ was only found in the familial and sporadic cases [26]. There is also a likely role for Aβ_pE3-42_ in intra-neuronal AD toxicity. A study that expressed Aβ_Q3-42_ under the Thy-1 promoter in mice (glutamine was used instead of glutamate because it is a better substrate for pyroglutamate conversion, [15]) showed increased levels of intra-neuronal Aβ_pE3-42,_ severe neurological impairment and loss of Purkinje cells [27]. Furthermore, Aβ_pE3-42_ expressing mice which display neuronal loss [28], when crossed into a tau KO background were almost completely protected against neuronal loss, establishing a functional connection between pGluAβ and tau [25]. Moreover, transgenic mouse models of AD that develop more severe pathology, as measured by the appearance of early neurological phenotypes and amyloid plaque deposition, tend to have high levels of Aβ_pE3-42_ [14].

Over-expression or reduction of QC has also been shown to exacerbate or rescue behavioural phenotypes and plaque pathology in an AD mouse model [29]. Interestingly, QC KO mice showed a reduction in both Aβ_pE3-42_ and Aβ_1-42_ levels, again supporting the idea that Aβ_pE3-42_ plays a role in seeding Aβ_1-42_ [29]. The data also demonstrate the importance of QC, and suggest that a reduction of QC might be a promising therapeutic strategy.

Many signaling pathways/molecules are conserved between flies and humans, and QC is 1 of them. *Drosophila* has 2 QCs – Drome QC and isoDrome QC, which have different subcellular locations [30]. IsoDrome QC more closely resembles the mammalian homologue [30]. Interestingly, treatment of Aβ_Q3-42_ transgenic flies with a QC inhibitor led to reduced Aβ_pE3-42_ levels [18], highlighting the usefulness of *Drosophila* to investigate the molecular pathogenicity of Aβ_pE3-42._ Several labs have generated fly models that express various Aβ peptides [31–33]. Aβ_Q3-42_ fly models are available, but have not been fully characterized or utilized to test the “seeding hypothesis”.

In this study, we characterized a *Drosophila* model of Aβ_pE3-42_ toxicity in the fruit-fly. Expression specifically in adult fly neurons led to behavioural dysfunction and shortened lifespan. Expression of the Aβ_pE3-42_ constitutively in the eyes led to disorganised ommatidia, which was ameliorated by neprilysin2. Furthermore, we show for the first time that neprilysin2 was able to degrade pyroglutamate Aβ.

Several recent studies have suggested that Aβ_pE3-42_ can act as a seed for Aβ1-42, and such a role has been demonstrated in vitro. Aβ_pE3-42_ has been shown to increase the amount of metastable low-n Aβ_1-42_ oligomers in vitro [25]. Furthermore, peri-hippocampal injection of Aβ_pE3-42_ into APPswe/NOS2-/- AD mice led to the presence of both Aβ_pE3-42_ and conventional Aβ plaques_,_ which the authors mention was hardly seen in sham injected AD mice or WT mice injected with Aβ_pE3-42_ [25]. However, the direct effect of Aβ_pE3-42_ on Aβ_1-42_ in vivo remains to be assessed, because the genetic background of the AD mouse lines are mutant for several genes that may well be affected by Aβ_pE3-42._ Furthermore, to control for the specificity of the Aβ_pE3-42_ species in causing enhanced plaque formation, an important control of peri-hippocampal injection of Aβ_1-42_ into the AD mice is missing from their studies.

We have utilized the *Drosophila* model to our advantage by expressing multiple transgenes at the same time to test this seeding hypothesis in vivo, and thus determine whether Aβ_pE3-42_ could be a target for therapeutic intervention, and/or a diagnostic marker. We found that total Aβ_1-42_ levels and toxicity are greatly increased when Aβ_pE3-42_ is co-expressed. These data suggest that Aβ_pE3-42_ is able to stabilise Aβ_1-42_ in vivo.

## Materials and methods

### Fly stocks and maintenance

All fly stocks were maintained either at 25 °C or 28 °C on a 12:12-h light:dark cycle at constant humidity on a standard sugar-yeast (SY) medium (15gl^-1^ agar, 50 gl^-1^ sugar, 100 gl^-1^ autolysed yeast, 100gl^-1^ nipagin and 3 ml l^-1^ propionic acid). Adult-onset, neuronal-specific expression of Aβ peptide was achieved by using the elav GeneSwitch (elavGS)-UAS system. ElavGS was derived from the original elavGS 301.2 line [34] and obtained as a generous gift from Dr H. Tricoire (CNRS, France), GMR driver was from Bloomington stock centre. UAS-Aβ1-42 line was obtained from Dr D. Crowther [35]. UAS- Aβ_Q3-42_ line has been previously described [36], briefly the rat pre pro-enkephalin signaling sequence was cloned upstream Aβ_Q3-42_ and put into the EcoR1 site of the pUAST vector, the construct then gets processed via prohormone convertases and glutaminyl cyclase to generate Aβ_pE3-42_ [17, 37] (Additional file 1: Figure S1). GMR, elavGS and UAS-lines used in all experiments were backcrossed six times into the *w* ^1118^ genetic background. Expression by elavGS was induced by treatment with mifepristone (RU486; 200 μM) added to the standard SY medium. In the absence of mifepristone (RU486; -RU), the transgene remains transcriptionally silent. Following treatment with RU486, Aβ_Q3-42_ peptide is expressed.

### Lifespan analyses

For all experiments, flies were raised at a standard density on standard SY medium in 200 mL bottles. Two days after eclosion once-mated females were transferred to experimental vials containing SY medium with or without RU486 (200 μM) at a density of 10 flies per vial (120 flies per genotype were used in Fig. 2, and 150 flies per genotype were used in Fig. 4). Deaths were scored almost every other day and flies were transferred to fresh food. Data are presented as survival curves and statistical analysis was performed using log-rank tests to compare survival of groups.

### Negative geotaxis assays

Climbing assays were performed at 25 °C according to previously published methods [31]. Climbing was analysed every 2–3 days post-RU486 treatment. Fifteen adult flies were placed in a vertical column, then their rate of climb to the top of the column was analysed. Flies reaching the top (12 cm) and flies remaining at the bottom of the column after a 30 s period were counted separately, and 3 trials were performed for each experiment. Scores recorded were the mean number of flies at the top (n_top_), the mean number of flies at the bottom (n_bottom_) and the total number of flies assessed (n_tot_). A performance index (PI) defined as ½(n_tot_ + n_top_ - n_bottom)/_ n_tot_) was calculated. Data are presented as the mean PI ± SEM obtained in 3 independent experiments for each group, and analyses of variances (ANOVA) were performed using JMP software.

### Quantification of Aβ peptide by ELISA

Quantification of Aβ was carried out as previously described [38]. To extract total Aβ, 5 *Drosophila* heads were homogenised in 50 μl GnHCl extraction buffer (5 M Guanidine HCl, 50 mM Hepes pH 7.3, protease inhibitor cocktail (Sigma, P8340) and 5 mM EDTA), centrifuged at 21,000 g for 5 min at 4 °C, and cleared supernatant retained as the total fly Aβ sample. Alternatively, for soluble and insoluble pools of Aβ, 25 fly heads were homogenised in 50 μl tissue homogenisation buffer (250 mM sucrose, 20 mM Tris base, 1 mM EDTA, 1 mM EGTA, protease inhibitor cocktail (Sigma) then mixed further with 50 μl diethyl acetate (DEA) buffer (0.4 % DEA, 100 mM NaCl and protease inhibitor cocktail). Samples were centrifuged at 135,000 g for one hour at 4 °C (Beckman Optima^TM^ Max centrifuge, TLA120.1 rotor), and supernatant retained as the cytosolic, soluble Aβ fraction. Pellets were resuspended in 200 μls ice-cold formic acid (FA; 70 %), and sonicated. Samples were re-centrifuged at 135,000 g for one hour at 4 °C, then 100 μl of supernatant diluted with 1 ml FA neutralisation buffer (1 M Tris base, 0.5 M Na_2_HPO_4_, 0.05 % NaN_3_) and retained as the insoluble, formic acid-extractable Aβ fraction. Aβ content was measured using the hAmyloid β42 ELISA kits, X-42 and N3pE-42 (IBL INTERNATIONAL). N of 3 or 4 individual samples were diluted in sample/standard dilution buffer and ELISA performed according to the manufacturers’ instructions. Protein extracts were quantified using the Bradford protein assay (Bio-Rad protein assay reagent; Bio-Rad laboratories (UK) Ltd) and the amount of Aβ in each sample expressed as a ratio of the total protein content (pmol/g total protein).

### Western blotting

For total Aβ extraction, we used a procedure previously described [11]. 20 heads per biological replicate were homogenized in 100 μL of 70 % formic acid. Samples were centrifuged at 16,000 *g* for 20 min at room temperature. The supernatant was collected and evaporated using a SpeedVac. The pellet was resuspended in 100 μL 2× LDS containing reducing agent (Invitrogen) and homogenized by sonication (10 pulses). Samples were then boiled at 100 °C for 10 min and 15 μL of each sample were used for western blotting to determine total Aβ levels. For LDS/SDS oligomer Aβ extraction, 20 heads per biological replicate were homogenized in 100 μL 2× LDS containing reducing agent (Invitrogen). Samples were incubated on ice for 30 min and then boiled at 100 °C for 10 min. 15 μL per sample were used for western blotting to evaluate LDS/SDS-stable Aβ oligomers. Proteins were separated on 16.5 % Tris-Tricine Criterion gels (Biorad) blotted onto nitrocellulose membranes. Membranes were incubated in a blocking solution containing 5 % milk proteins in TBST for 1 h at room temperature, then probed with primary antibody diluted in TBST + 5 % BSA overnight at 4 °C. 82E1 Aβ1-42 Antibody was from Takara, used at 1 in 100 dilution.

For pJNK western blot analyses, total protein was extracted from 5 fly heads in 30 μl 2 × LDS buffer containing reducing agent (Invitrogen). Membranes were incubated in a blocking solution containing 5 % BSA in TBST for 1 h at room temperature, then probed with primary antibody diluted in TBST + 5 % BSA overnight at 4 °C. mouse monoclonal phospho-SAPK/JNK (T183/Y185) antibody was from cell signaling, used at 1 in 1000 dilution. Rabbit polyclonal actin antibody was from abcam and used at 1 in 1000 dilution.

### Quantitative RT-PCR

Total RNA was extracted from 20 to 25 fly heads using TRIzol (GIBCO) according to the manufacturers’ instructions. The concentration of total RNA purified for each sample was measured using an *Eppendorf biophotometer.* 1 μg of total RNA was then subjected to DNA digestion using DNAse I (Ambion), immediately followed by reverse transcription using the Superscript II system (Invitrogen) with oligo(dT) primers. Quantitative PCR was performed using the PRISM 7000 sequence-detection system (Applied Biosystems), SYBR Green (Molecular Probes), ROX Reference Dye (Invitrogen), and Hot StarTaq (Qiagen, Valencia, CA) by following manufacturers’ instructions. Each sample was analysed at a minimum in triplicate with both target gene (Aβ_Q3-42_ or NEP2) and control gene (RP49) primers in parallel. The primers for the Aβ transgenes were directed to the 5’ end and 3’ end of the Aβ coding sequence: forward CGACATGACTCAGGTTATGAAGTT; reverse GACAACGCCCACCAT Neprilysin2 primers are, forward ACGAGGTCAACTGGATGGAC and reverse GTCGAGCTTGGCGTAGTAGG. RP49 primers were as follows: forward ATGACCATCCGCCCAGCATCAGG; reverse ATCTCGCCGCAGTAAACG.

### Eye phenotype

Eye images of 2/3-day-old female flies expressing Aβ_pE3-42_ under the control of the GMR-Gal4 driver at 28 °C were taken. Nail polish imprint of the external eye was carried out as previously described. For adult eye transverse sections, adult heads were fixed, dehydrated, sectioned (10 microns thick) and stained with Harry’s hematoxylin. To investigate the eye phenotype of double transgenic Aβ_1-42;_ Aβ_Q3-42_ flies, we kept the flies at 25 °C to minimize Aβ_pE3-42_ eye phenotype. Images were taken with ZEISS Axioskop2 plus microscope. The eye phenotype was quantified by assigning numbers, from zero to 2 to individual flies chosen at random. Normal looking eyes were given zero, flies with moderate eye phenotype were assigned 1, and flies with strong eye phenotype were given 2, (*N* = 5 − 6 flies per genotype). The scoring was carried out blind by 2 independent researchers.

### Statistical analyses

For lifespan analyses, log-rank tests were used to assess for statistical differences. Eye phenotype was presented as means ± SEM, and statistically assessed by Student’s *t* test. Other data are presented as means ± SEM obtained in at least 3 independent experiments, and differences between means were assessed by either Student’s *t* test or 2-way analysis of variance (ANOVA) using JMP (version 12.0) software (SAS Institute, Cary, NC, USA).

## Results

### Pyroglutamate Aβ (Aβ_pE3-42_) expression can be induced in the adult *Drosophila* nervous system

**Fig. 1:**
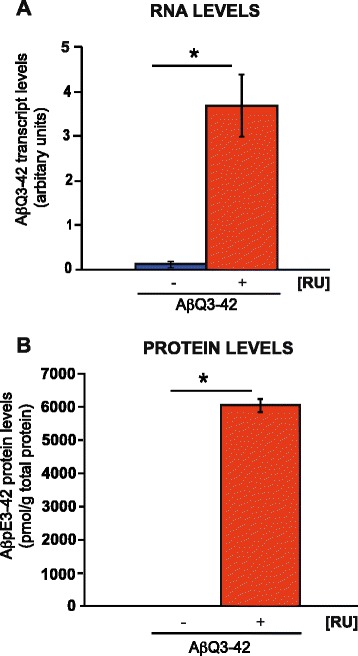
Pyroglutamate Aβ expression in the *Drosophila* nervous system. **a** RNA levels were quantified at 7 days post-RU486 treatment and (**b**) Aβ_pE3-42_ protein levels were quantified at 21 days post-RU486 treatment. Data are presented as means ± SEM and were analysed by Student *t* test, *P < 0.001 comparing Aβ RNA and protein expression in RU486-treated elavGS/UAS-Aβ_Q3-42_ flies to their –RU486 controls

A fly model that expresses Aβ_E3-42_ has been described [39], however, we are utilising a previously generated Aβ_Q3-42_ transgenic fly model for this study, since glutamine is a better substrate for pyroglutamate conversion than glutamate [14]. Aβ_Q3-42_ fly models have been generated but little characterized [18, 36]. To ensure that these flies could express Aβ_Q3-42,_ we drove expression of the Aβ_Q3-42_ transgene in adult neurons with the inducible pan-neuronal driver, elav GeneSwitch (elavGS) [31, 34]. We measured RNA levels of Aβ_Q3-42_ flies in adult neurons, after treating elavGS;UAS-Aβ_Q3-42_ flies with the activator mifepristone (RU486) for 7 days, starting at 2 days post-eclosion (Fig. 1a). We found a significant increase in Aβ_Q3-42_ transcripts in RU486-treated elavGS;UAS-Aβ_Q3-42_ flies in comparison to untreated flies (Fig. 1a). Furthermore, we confirmed that the flies generate pyroglutamate-modified Aβ by measuring Aβ_pE3-42_ protein levels specifically. Aβ_pE3-42_ protein levels in adult neurons of elavGS;UAS-Aβ_Q3-42_ flies treated with RU486 for 21 days, starting at 2 days post-eclosion were significantly increased in comparison to untreated flies (Fig. 1b). These data demonstrate that Aβ_pE3-42_ can be successfully generated in the flies.

### Expression of Aβ_pE3-42_ causes shortened lifespan, neuronal dysfunction, disorganised eye phenotype, and activates JNK in *Drosophila*

**Fig. 2:**
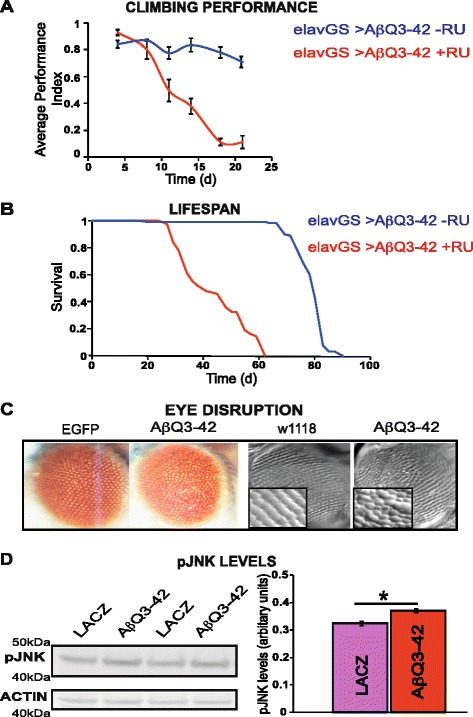
Expression of Aβ_pE3-42_ causes locomotor dysfunction, shortened lifespan, eye disruption, and JNK activation in *Drosophila*. **a** Climbing ability of elavGS**/**UAS-Aβ_Q3-42_ flies on + and – RU486 SY medium were assessed at the indicated time-points. Data are presented as the average performance index (PI) ± SEM and were compared using 2-way ANOVA (number of independent tests (n) = 3 *P < 0.001 (**b**) Expression of Aβ_pE3-42_ in adult neurons shortens lifespan. Survival curves are depicted and data were compared using the log-rank test, *P < 0.001 comparing elavGS/UAS-Aβ_Q3-42_ + RU flies to -RU flies. **c** Expression of Aβ_pE3-42_ causes a neurodegenerative eye phenotype. First 2 images from left to right are light microscopy images and latter 2 images are nail varnish imprints of eyes (magnification is 25×, and 40× objectives for close up images). From left to right, GMR-GAL4/UAS-EGFP, GMR-GAL4/+;UAS-Aβ_Q3-42_/+, GMR-GAL4/+, GMR-GAL4/+;UAS-Aβ_Q3-42_/+. Note compressed and fused ommatidia in Aβ_Q3-42_ expressing flies in comparison to control flies (GMR-GAL4/UAS-EGFP or GMR-GAL4/+). Flies were grown at 28 °C. **d** Expression of Aβ_pE3-42_ increases the levels of phosphorylated JNK. Data are presented as means ± SEM and were analysed by Student *t* test, *P < 0.05 comparing GMR-GAL4/+;UAS-Aβ_Q3-42_/+ flies to control GMR-GAL4/UAS-LACZ flies

To determine whether expression of Aβ_pE3-42_ in neurons is toxic, we used the elavGS driver to express Aβ_pE3-42_ peptide in adult neurons, and measured the effects on behaviour. Impaired geotaxis is a behavioural measure of neuronal dysfunction and can be assessed using a climbing assay [31]. elavGS;UAS-Aβ_Q3-42_ flies were treated with RU486 starting at 2 days post-eclosion, and their climbing ability was subsequently recorded. Flies expressing Aβ_pE3-42_ displayed substantially increased rate of decline in negative geotaxis with age in comparison to the –RU or driver (elavGS) alone control flies (Fig 2a and Additional file 2: Figure S2).

We also measured the effects of Aβ_pE3-42_ on lifespan in comparison to the –RU control flies, by treating elavGS,UAS-Aβ_Q3-42_ flies with RU486 starting at 2 days post-eclosion and recording their subsequent survival. Expression of Aβ_pE3-42_ in adult neurons significantly shortened median (54 %) and maximum lifespan (31 %) in comparison to control elavGS,UAS-Aβ_Q3-42_ -RU flies (Fig. 2b).

To assess the effects of Aβ_pE3-42_ on neurodegeneration, we expressed Aβ_pE3-42_ constitutively in the fly eye using the GMR-GAL4 driver and observed the effect on the organization of the ommatidia, an assay that has been used extensively to characterise fly models of neurodegenerative diseases [35]. Expression of Aβ_pE3-42_ caused disorganisation of the ommatidia in these flies, presenting with eye roughness and fused ommatidia in comparison to flies expressing EGFP, and w1118 control flies (Fig. 2c).

The JNK/SAPK signaling pathway which influences cell death is activated by Aβ, and has been suggested to contribute to Aβ mediated cell death in fly models expressing Aβ_1-42_ [40, 41]. To determine whether Aβ_pE3-42_ expressing flies are also capable of activating JNK, we measured the levels of phosphorylated JNK as a read-out in the flies, by western blot analyses. We found a significant increase in the level of phosphorylated JNK in flies expressing Aβ_pE3-42_ (GMR-GAL4/+; Aβ_Q3-42_/+) in comparison to control flies expressing LACZ (GMR-GAL4/LACZ), 6 days post-eclosion (Fig. 2d).

### Co-expression of neprilysin2 suppresses the toxicity of Aβ_pE3-42_ expressing flies

**Fig. 3:**
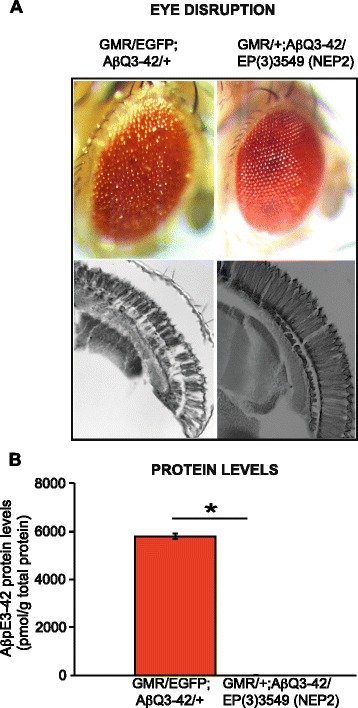
**a** Co-expression of neprilysin2 suppresses the toxicity of Aβ_pE3-42_ expressing flies. Top, light microscopy images and bottom, transverse sections. From left to right, GMR-GAL4/UAS-EGFP;UAS-Aβ_Q3-42_/+ and GMR-GAL4/UAS-EGFP;UAS-Aβ_Q3-42_/NEP2 (EP(3)3549). Flies were grown at 28 °C. **b** Neprilysin2 significantly reduces Aβ_pE3-42_ protein levels, *P < 0.001_._ Aβ_pE3-42_ levels were quantified by ELISA. Data are presented as means ± SEM and were analysed by Student *t* test

Neprilysin (NEP) and its close homologue Neprilysin2 (Nep2) are candidate Aβ degrading enzymes, and regulate amyloid protein levels in AD [42–44]. We next determined whether the fly orthologue, *nep2,* which has been shown previously to significantly reduce Aβ_1-42_ levels and toxicity, is able to similarly ameliorate Aβ_pE3-42_ induced toxicity. We made use of the rough eye/disorganised ommatidia phenotype, and found that co-expression of NEP2 using the EP(3)3549 *Drosophila* strain, with the GMR-GAL4 driver almost completely suppressed both the external disorganised ommatidia and internal retinal degeneration of the Aβ_pE3-42_ expressing flies (Fig. 3a). We confirmed that the EP(3)3549 strain had a significant expression of NEP2 levels by RTPCR (Additional file 3: Figure S3A).

### Co-expression of neprilysin2 reduces Aβ_pE3-42_ protein levels

To understand the mechanism by which NEP2 ameliorates the Aβ_pE3-42_ eye phenotype, we measured total Aβ load, and Aβ_pE3-42_ protein levels specifically in the flies, 7 days post-eclosion. Interestingly, we found by ELISA analyses that Aβ load and importantly, Aβ_pE3-42_ levels were significantly reduced in flies co-expressing Aβ_pE3-42_ and NEP2 in comparison to flies co-expressing Aβ_pE3-42_ and EGFP as a control (Additional file 3: Figure S3B and Fig. 3b). Furthermore, the reduction we see at the protein level is not due to reduced RNA levels, since this reduction was not observed in the RNA by RTPCR analyses, 7 days post eclosion (Additional file 3: Figure S3C). These data demonstrate a major role of NEP2 in ameliorating Aβ_pE3-42_ induced toxicity, by reducing Aβ_pE3-42_ protein levels.

### Aβ_pE3-42_ is more toxic than Aβ_1-42_ in *Drosophila*

**Fig. 4:**
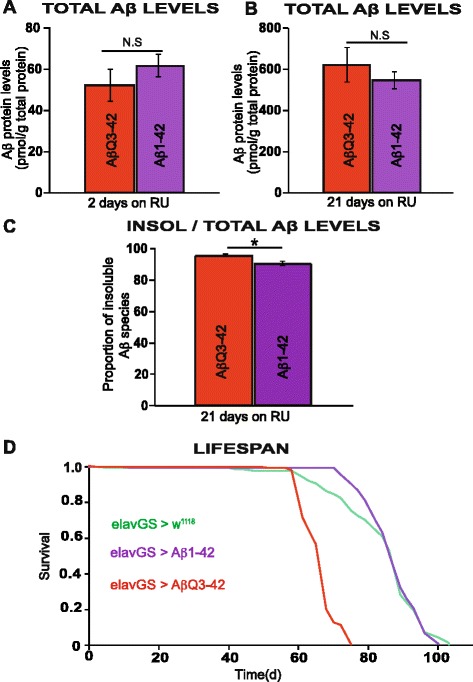
Aβ_pE3-42_ is more toxic than Aβ_1-42_ in *Drosophila.* Following treatment with RU486, Aβ_1-42_ and Aβ_pE3-42_ peptides were expressed at similar levels, quantified at 2 days and 21 days post-RU486 treatment (**a**) and (**b**) respectively. **c** A significant increase in the amount of insoluble to total Aβ protein levels in Aβ_pE3-42_ expressing flies in comparison to Aβ_1-42_ expressing flies was observed when quantified at 21 days post-RU486 treatment. Data are presented as means ± SEM and were analysed by Student’s *t* test, *P* < 0.01. **d** Expression of Aβ_pE3-42_ specifically in adult neurons shortens lifespan significantly relative to both Aβ_1-42_ and w1118 control. Survival curves are depicted and data were compared using the log-rank test. *P < 0.001 comparing elavGS/UAS-Aβ_Q3-42_ + RU flies to elavGS/+ and UAS-Aβ_1-42_ /+;elavGS/+ +RU flies

Data from mouse models have indicated that the appearance of Aβ_pE3-42_ correlates with increased pathogenicity [45]. To determine whether Aβ_pE3-42_ peptide was more toxic in comparison to Aβ_1-42_, we needed 2 lines with comparable levels of Aβ peptide. We expressed the peptides with the elavGS driver line, by treating Aβ_Q3-42_ and Aβ_1-42_ transgenic flies independently with RU486 starting at 2 days post-eclosion, for 2 days and 21 days, and measured Aβ protein levels in adult neurons, taking advantage of an ELISA kit that recognizes an epitope in the middle of both Aβ_pE3-42_ and Aβ_1-42_ peptides. We found similar levels of total Aβ protein in the Aβ_pE3-42_ and Aβ_1-42_ expressing flies (Fig. 4a and b). However, the solubility/aggregation propensity of Aβ differed between the Aβ_pE3-42_ and Aβ_1-42_ expressing flies, with Aβ_pE3-42_ expressing flies having a significantly increased ratio of insoluble to total Aβ in comparison to Aβ_1-42_ expressing flies (Fig. 4c). Aβ_pE3-42_ expressing flies also suffered increased toxicity, because expression of Aβ_pE3-42_ in adult neurons significantly shortened median (27 %) and maximum (23 %) lifespan in comparison both to control w1118;;elavGS and Aβ_1-42_ expressing flies (Fig. 4d), demonstrating a more toxic effect of the Aβ_pE3-42_ peptide.

### pGluAβ increases accumulation of Aβ in vivo

**Fig. 5:**
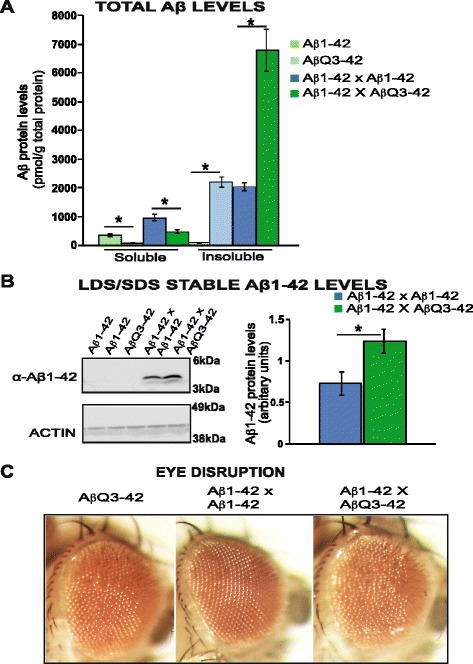
pGluAβ increases accumulation of Aβ in vivo, and exacerbates toxicity (**a**). Flies expressing Aβ_1-42_ had significantly more soluble Aβ, *P* <0.05 but less insoluble Aβ, *P* <0.0001 in comparison to flies expressing Aβ_pE3-42_. Flies co-expressing Aβ_1-42_ and Aβ_pE3-42_ had significantly more insoluble Aβ, *P* <0.01 but less soluble Aβ *P* <0.05 Aβ in comparison to flies co-expressing Aβ_1-42_ and Aβ_1-42_. Data are presented as means ± SEM and were analysed by Student’s *t*-test. **b** There was a significant increase in Aβ_1-42_ accumulation in flies co-expressing Aβ_1-42_;Aβ_pE3-42_ in comparison to flies expressing either a single copy of Aβ_1-42_ (expressing one single copy of Aβ_1-42_ does not accumulate enough protein levels for quantification by western blot analyses), or flies expressing 2 copies of Aβ_1-42,_ *P* < 0.05_._ Data are presented as means ± SEM and were analysed by Student’s *t* test. GMR-GAL4/+;UAS-Aβ_1-42_/+, GMR-GAL4/+;UAS-Aβ_Q3-42_/+, GMR-GAL4/UAS-Aβ_1-42_;UAS-Aβ_1-42_/+ and GMR-GAL4/+;UAS-Aβ_1-42_/UAS-Aβ_Q3-42_ flies were used for (**a**) and (**b**). **c**. Co-expression of Aβ_1-42_ and Aβ_pE3-42_ led to a disorganized eye phenotype that was absent in flies co-expressing Aβ_1-42_ and Aβ_1-42,_ and worse than in Aβ_pE3-42_ expressing flies. From left to right, GMR-GAL4/+;UAS-Aβ_Q3-42_/+, GMR-GAL4/UAS-Aβ_1-42_;UAS-Aβ_1-42_/+ and GMR-GAL4/+;UAS-Aβ_1-42_/UAS-Aβ_Q3-42_ flies. Flies were grown at 25 °C

Aβ_pE3-42_ has been shown to increase the amount of metastable low-n Aβ_1-42_ oligomers in vitro [25]. To study in vivo, the seeding effect of Aβ_pE3-42_, we co-expressed Aβ_pE3-42_ and Aβ_1-42_ peptides directly and compared the effects to those seen in flies expressing Aβ_1-42_. We expressed Aβ_1-42_ or Aβ_pE3-42_ constitutively with the GMR-GAL4 driver line, and measured at 2−3 days post-eclosion total protein levels of flies expressing either peptide. We found a substantial increase in insoluble Aβ levels and a significant decrease in soluble Aβ detected in Aβ_pE3-42_ expressing flies in comparison to Aβ_1-42_ expressing flies with ELISA (Fig. 5a). There was also a significant increase in total Aβ levels in Aβ_1-42_; Aβ_pE3-42_ expressing flies in comparison to Aβ_1-42_;Aβ_1-42_ expressing flies (Fig. 5a and Additional file 4: Figure S4). Furthermore, we found a shift in Aβ solubility in Aβ_1-42_; Aβ_pE3-42_ expressing flies, with a significant decrease in soluble Aβ and a substantial increase in insoluble Aβ in comparison to Aβ_1-42_;Aβ_1-42_ expressing flies (Fig. 5a).

We next investigated the effect of co-expressing Aβ_1-42_; Aβ_pE3-42_ specifically on Aβ_1-42_ stability, and whether this contributed to the increased total Aβ levels. We measured Aβ_1-42_ levels by western blot, by selecting an antibody that detects Aβ_1-42_, but not Aβ_pE3-42_ (Fig. 5b and Additional file 5: Figure S5). Flies co-expressing Aβ_1-42_; Aβ_pE3-42_ had increased levels of Aβ_1-42_ levels in comparison to flies expressing a single copy of Aβ_1-42_, which do not accumulate enough protein levels for quantification by western blot analyses (Fig. 5a and Additional file 5: Figure S5). However, Aβ_1-42_ was specifically detected in flies containing 2 copies of the Aβ_1-42_ transgene (Aβ_1-42_;Aβ_1-42_), confirming protein expression of this line with the antibody (Fig. 5b). Interestingly, the flies co-expressing Aβ_1-42_;Aβ_1-42_ significantly expressed lower levels of Aβ_1-42_ in comparison to flies co-expressing Aβ_1-42_; Aβ_pE3-42_ (Fig. 5b).

### pGluAβ enhances Aβ toxicity

Since Aβ_pE3-42_ increased the stability of Aβ_1-42_, we assessed whether it also increased the toxicity of Aβ_1-42_, using the rough eye/disorganised ommatidia phenotype. Flies co-expressing Aβ_1-42_; Aβ_pE3-42,_ but not those expressing Aβ_1-42_;Aβ_1-42_ presented with disorganised ommatidia, 2 days post eclosion, and this phenotype was stronger than in flies expressing Aβ_Q3-42_ alone (Fig. 5c and Additional file 6: Figure S6) suggesting that Aβ_pE3-42_ enhances the toxicity of Aβ_1-42_.

Collectively, these data suggest that Aβ_pE3-42_ is able to increase the stability of the Aβ_1-42_ peptide and exacerbate its toxicity in vivo.

## Discussion

Aβ_pE3-42_ is increasingly thought to play a pivotal role in the pathogenesis of Alzheimer’s disease [14]. Although previous studies in vitro have suggested that Aβ_pE3-42_ acts as a seed for Aβ stability, and some correlative work has been done in vivo [25], this seeding behaviour and its consequences have not been examined in vivo. Our study demonstrates that Aβ_pE3-42_ increases the levels of Aβ_1-42_, presumably by increasing its stability, and that it enhances toxicity of the Aβ_1-42_ peptide, as observed in flies co-expressing Aβ_pE3-42_ and Aβ_1-42_ in comparison to flies expressing 2 copies of Aβ_1-42_.

First, we characterized the model. Expression of the Aβ_pE3-42_ peptide specifically in adult fly neurons led to behavioural dysfunction and shortened lifespan, and constitutive expression in the eyes led to disorganised ommatida. Furthermore, we found that Aβ_pE3-42_ was able to activate the JNK signaling pathway, suggesting a role for this cell death activating pathway in Aβ_pE3-42_ mediated toxicity.

Interestingly, we found that we could ameliorate the Aβ_pE3-42_ toxicity by over expressing Neprilsyin2. Increasing the expression of several candidate in vivo Aβ degrading enzymes, such as NEP or Insulin degrading enzyme (IDE) have been shown to reduce the cerebral amyloid plaque burden observed in APP over-expressing mice [46]. However, direct interactions between Aβ_pE3-42_ and Neprilysin have not been investigated. We found that over-expression of *Drosophila* NEP2 was able to reduce Aβ_pE3-42_ levels and improve considerably the disorganised ommatidia, demonstrating for the first time that, although Aβ_pE3-42_ may aggregate more than Aβ_1-42_, NEP2 is capable of degrading pyroglutamate-modified Aβ.

Also, we found that these flies had an increase in the ratio of insoluble to total Aβ levels in comparison to flies expressing Aβ_1-42,_ confirming the propensity of Aβ_pE3-42_ to aggregate as previously described [14]. Furthermore, we found that Aβ_pE3-42_ was more toxic than Aβ_1-42_ peptide.

Another interesting finding from our analyses is the increase in Aβ levels observed in response to Aβ_pE3-42_. We found a general increase in total Aβ levels in flies co-expressing Aβ_pE3-42_ and Aβ_1-42,_ and there was also an increase in the ratio of insoluble Aβ to total Aβ levels in these flies. To determine what role Aβ_1-42_ might have in this, we measured Aβ_1-42_ levels specifically by western blot analyses. Interestingly, we found that total Aβ_1-42_ levels were increased when Aβ_pE3-42_ was co-expressed. Nussbaum et al. showed that Aβ peptides oligomerise by different pathways, and that the low-n oligomers of Aβ_pE3-42_ are structurally distinct from Aβ_1-42_, and far more cytotoxic, in the order of Aβ_pE3-42_ /Aβ_1-42_ > Aβ_pE3-42_ > Aβ_1-42_ [25]. We found that co-expressing 1 copy each of Aβ_1-42_ and Aβ_pE3-42_ increased the accumulation of Aβ_1-42_, and was more toxic than expressing 2 copies of Aβ_1-42_, indicating that Aβ_pE3-42_ is able to stabilise Aβ_1-42_ in a different manner to over expressing the Aβ_1-42_ peptide, perhaps by affecting its structure.

## Conclusions

We have tested and validated the Aβ_pE3-42_ seeding hypothesis and shown that indeed Aβ_pE3-42_ increases the levels of Aβ, and that Aβ_pE3-42_ enhances pathology in this AD model. These results raise Aβ_pE3-42_ as both a potential biomarker and new therapeutic target in AD. Furthermore, because *Drosophila* does not inherently express Aβ, the observation that expression of Aβ_pE3-42_ is able to cause toxicity independent of its effect on Aβ_1-42_ suggests that it is capable of initiating toxicity in other ways. It would be interesting to uncover downstream pathways that are modulated specifically by Aβ_pE3-42_, and the fly provides a powerful context for pursuing this question with genetic screens.

## Additional files

Additional file 1: Figure S1. Generation of Aβ_pE3-42._ The proenkephalin signaling peptide upstream of Aβ_Q3-42_ is cleaved by prohormone convertases, Aβ_Q3-42_ is then released, and glutaminyl cyclase catalyses its conversion to Aβ_pE3-42_. (PDF 271 kb)
Additional file 2: Figure S2. Expression of Aβ_pE3-42_ causes locomotor dysfunction. Climbing ability of elavGS**/**UAS-Aβ_Q3-42_and elavGS flies on + RU486 SY medium was assessed at the indicated time-points (see Materials & Methods). Expression of Aβ_pE3-42_ in adult neurons reduced climbing ability of the flies in comparison to control elavGS driver flies. Data are presented as the average performance index (PI) ± SEM and were compared using 2-way ANOVA (number of independent tests (n) = 3 *P* < 0.01. (PDF 306 kb)
Additional file 3: Figure S3. (A). Confirming the expression of Neprilysin 2 in the EP(3)3549 fly strain. There was a significant increase in *nep2* transcript levels in the flies expressing the EP(3)3549 EP element, in comparison to the control fly lines expressing LACZ. Data are presented as means ± SEM and were analysed by student’s *t*-test, *P* < 0.001. (B) Neprilysin2 significantly reduces Aβ protein levels, **P* < 0.001_**.**_ Aβ_X-42_ levels were quantified by ELISA. Data are presented as means ± SEM and were analysed by Student *t* test. (C) Neprilysin2 does not reduce Aβ_Q3-42_ RNA levels_._ There was a significant increase in Aβ_Q3-42_ RNA levels in flies co-expressing Aβ_Q3-42_ and NEP2 in comparison to flies expressing Aβ_Q3-42_ and EGFP, by quantitative RTPCR, *P* < 0.01, student’s *t*-test. GMR-GAL4 was used to drive expression of Aβ_Q3-42_ transgenic flies. (PDF 369 kb)
Additional file 4: Figure S4. pGluAβ increases accumulation of Aβ in vivo. Flies co-expressing Aβ_1-42_ and Aβ_pE3-42_ peptide, had significantly higher Aβ levels than flies co-expressing Aβ_1-42_ and Aβ_1-42_. Data are presented as means ± SEM and were analysed by student’s *t*-test, *P* < 0.01. GMR-GAL4 was used to drive expression of Aβ_1-42_ and Aβ_Q3-42_ transgenic flies. (PDF 417 kb)
Additional file 5: Figure S5. pGluAβ increases accumulation of Aβ1-42 in vivo. (A). Flies co-expressing Aβ_1-42_ and Aβ_pE3-42_ peptide, had substantially more Aβ1-42 levels than flies expressing Aβ_1-42_ alone. Aβ was not detected in flies expressing a single copy of Aβ_1-42_ because it does not accumulate enough protein. However, Aβ_1-42_ was detected in flies expressing 2 copies of Aβ_1-42_ (B), confirming protein expression in these flies with the antibody. Furthermore, to validate specificity, Aβ_1-42_ was not detected in flies expressing either a single copy or double copy of Aβ_pE3-42._ GMR-GAL4 was used to drive expression of Aβ_1-42_ and Aβ_Q3-42_ transgenic flies. (PDF 10727 kb)
Additional file 6: Figure S6. Blind scoring of disorganised eye phenotype. The data demonstrate a significant difference in the degree of severity of eye roughness in flies co-expressing Aβ_1-42_ and Aβ_pE3-42_ in comparison to flies expressing either 2 copies of Aβ_1-42_ (A), or Aβ_pE3-42_ only (B), **P* < 0.001 for both. Flies were grown at 25 °C. Data are presented as means ± SEM and were analysed by Student’s *t* test. GMR-GAL4 was used to drive expression of Aβ_1-42_ and Aβ_Q3-42_ transgenic flies. (PDF 303 kb)

## Abbreviations

AD: Alzheimer’s disease
APP: amyloid precursor protein
Aβ: Amyloid beta
elavGS: elav GeneSwitch
ER: Endoplasmic reticulum
IDE: Insulin degrading enzyme
JNK/SAPK: c-Jun N-terminal kinase/stress-activated protein kinase
Nep2: Neprilysin2
pGlu: Pyroglutamic
QC: glutaminyl cyclase
RU486, RU: mifepristone
