# Supplementary figures and images for: pGluAβ increases accumulation of Aβ in vivo and exacerbates its toxicity
Source: Acta Neuropathol Commun. 2016 Oct 7;4:109. doi: 10.1186/s40478-016-0380-x (PMC5055666; doi:10.1186/s40478-016-0380-x)

Fig S1

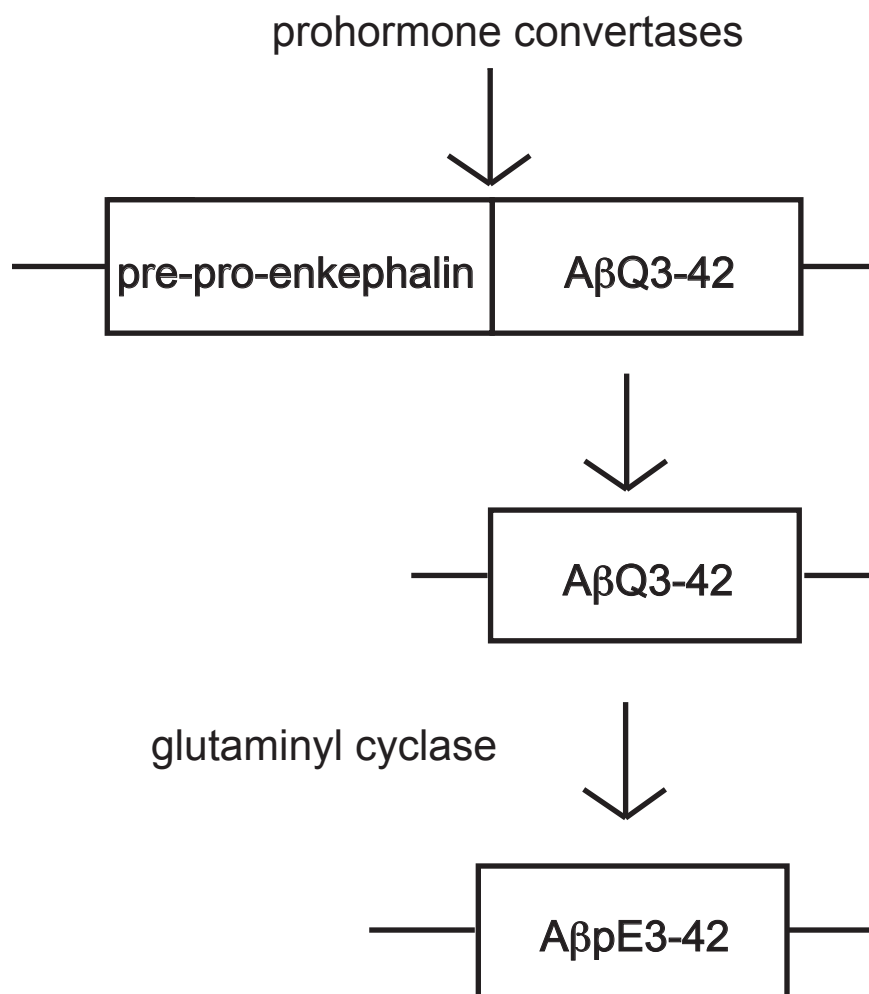

Supplement: Additional file 1: Figure S1. — Generation of AβpE3-42. The proenkephalin signaling peptide upstream of AβQ3-42 is cleaved by prohormone convertases, AβQ3-42 is then released, and glutaminyl cyclase catalyses its conversion to AβpE3-42. (PDF 271 kb) [file 40478_2016_380_MOESM1_ESM.pdf]

Fig S2

### CLIMBING PERFORMANCE

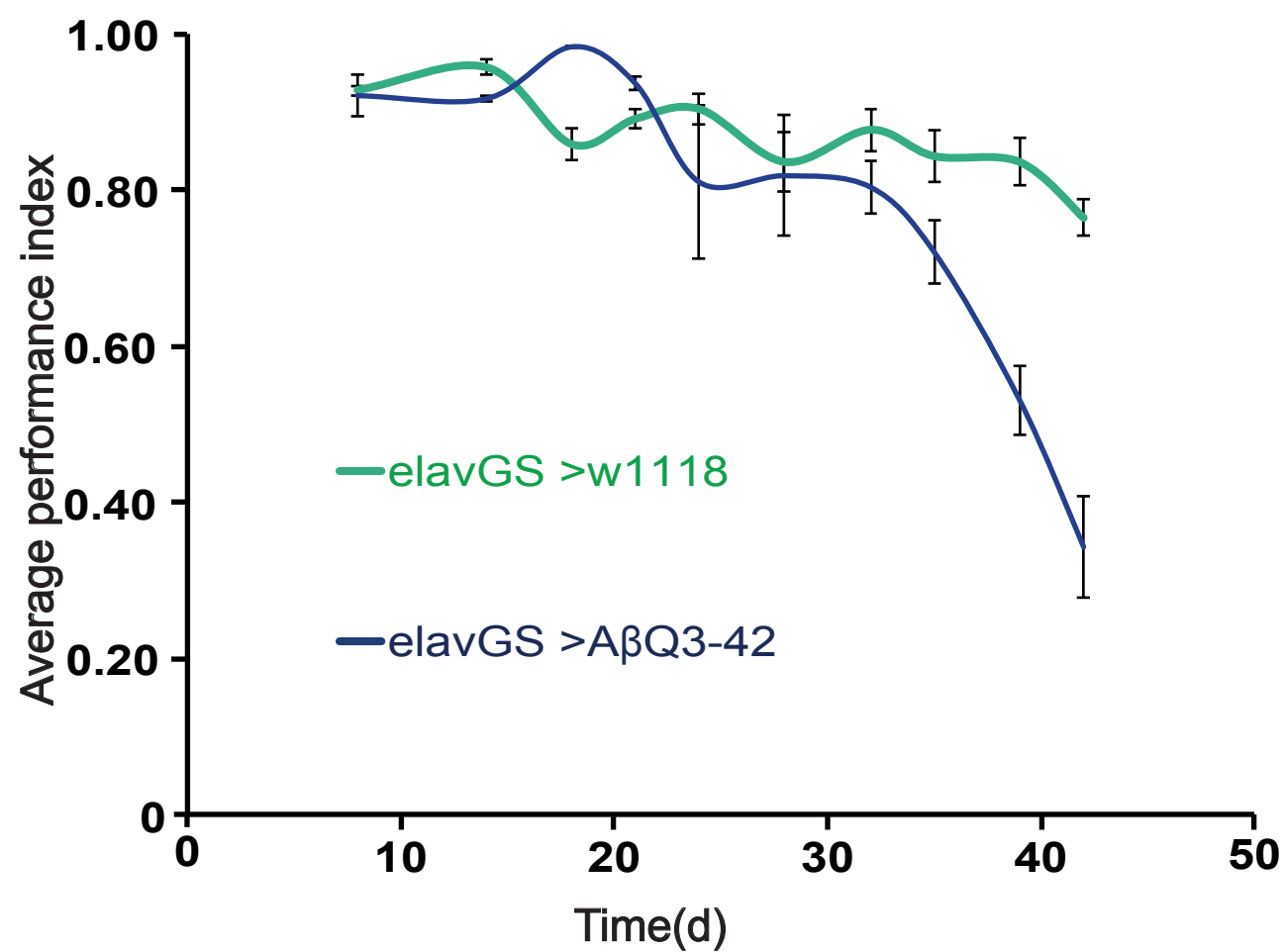

Supplement: Additional file 2: Figure S2. — Expression of AβpE3-42 causes locomotor dysfunction. Climbing ability of elavGS/UAS-AβQ3-42and elavGS flies on + RU486 SY medium was assessed at the indicated time-points (see Materials & Methods). Expression of AβpE3-42 in adult neurons reduced climbing ability of the flies in comparison to control elavGS driver flies. Data are presented as the average performance index (PI) ± SEM and were compared using 2-way ANOVA (number of independent tests (n) = 3 P < 0.01. (PDF 306 kb) [file 40478_2016_380_MOESM2_ESM.pdf]

**A****RNA LEVELS**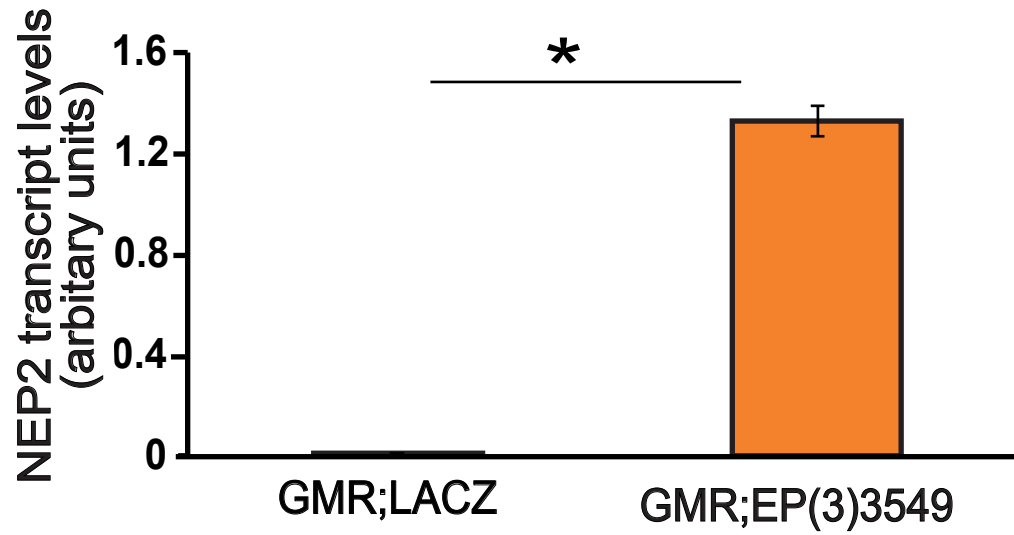**B****PROTEIN LEVELS**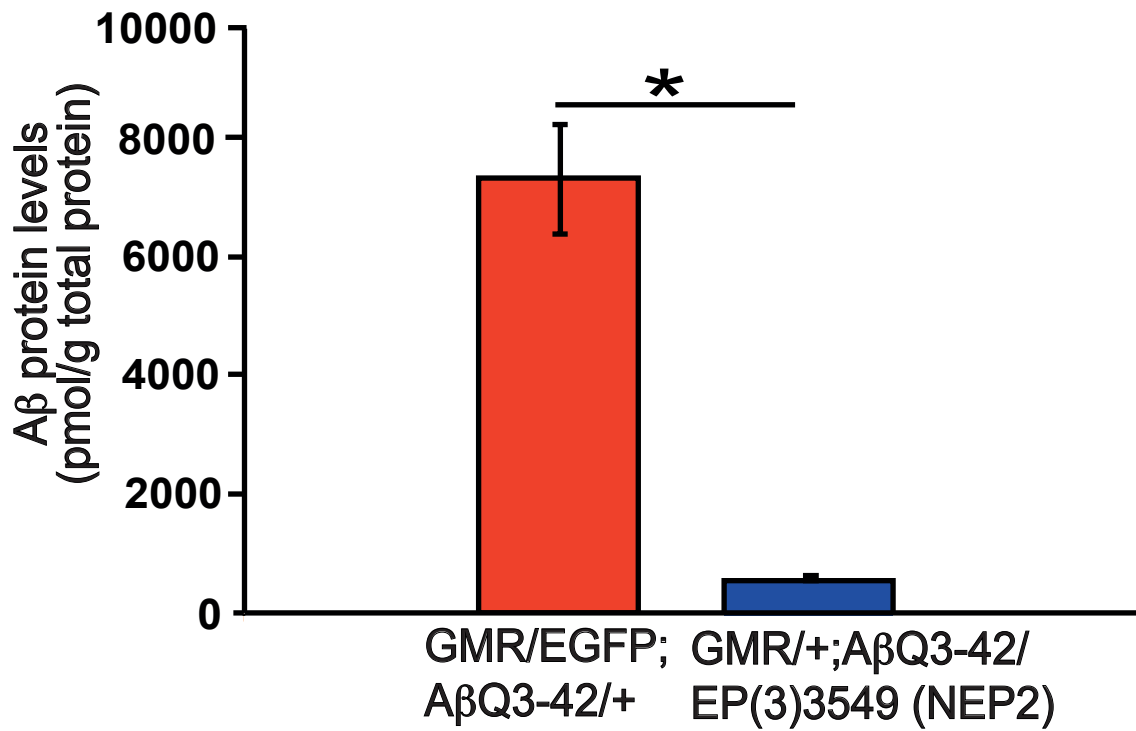**C****RNA LEVELS**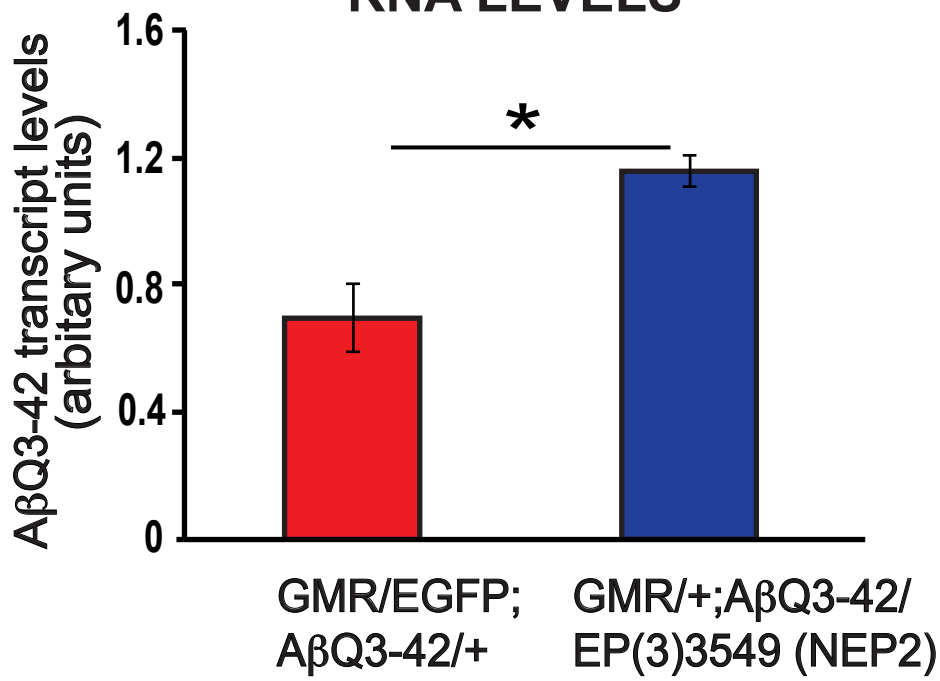

Supplement: Additional file 3: Figure S3. — (A). Confirming the expression of Neprilysin 2 in the EP(3)3549 fly strain. There was a significant increase in nep2 transcript levels in the flies expressing the EP(3)3549 EP element, in comparison to the control fly lines expressing LACZ. Data are presented as means ± SEM and were analysed by student’s t-test, P < 0.001. (B) Neprilysin2 significantly reduces Aβ protein levels, *P < 0.001. AβX-42 levels were quantified by ELISA. Data are presented as means ± SEM and were analysed by Student t test. (C) Neprilysin2 does not reduce AβQ3-42 RNA levels. There was a significant increase in AβQ3-42 RNA levels in flies co-expressing AβQ3-42 and NEP2 in comparison to flies expressing AβQ3-42 and EGFP, by quantitative RTPCR, P < 0.01, student’s t-test. GMR-GAL4 was used to drive expression of AβQ3-42 transgenic flies. (PDF 369 kb) [file 40478_2016_380_MOESM3_ESM.pdf]

Fig S4

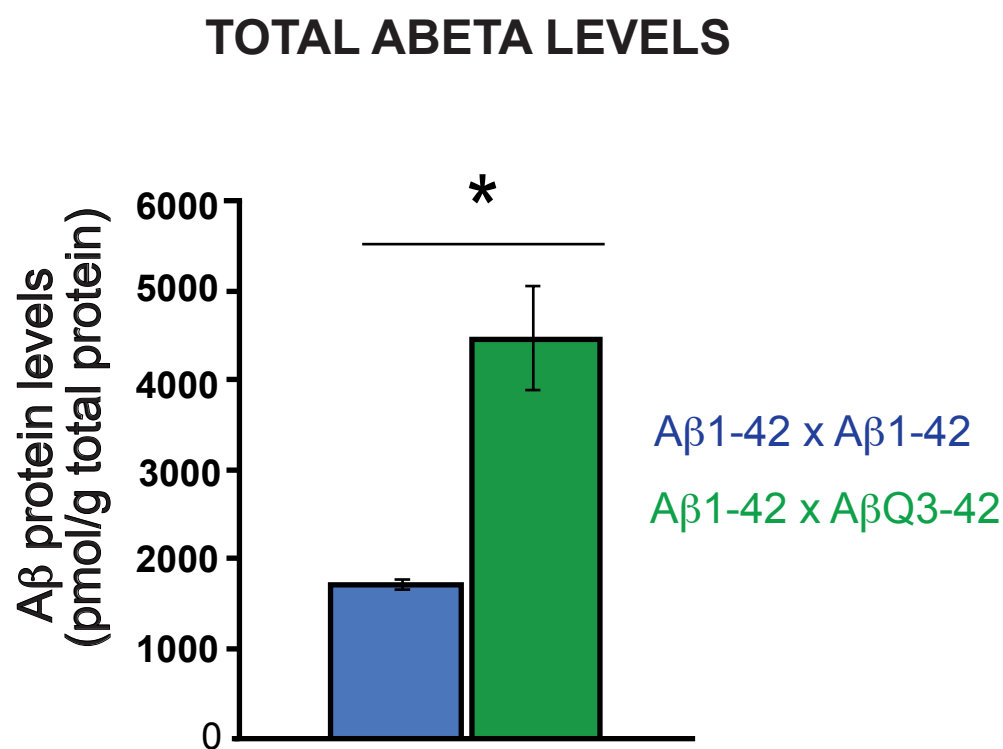

Supplement: Additional file 4: Figure S4. — pGluAβ increases accumulation of Aβ in vivo. Flies co-expressing Aβ1-42 and AβpE3-42 peptide, had significantly higher Aβ levels than flies co-expressing Aβ1-42 and Aβ1-42. Data are presented as means ± SEM and were analysed by student’s t-test, P < 0.01. GMR-GAL4 was used to drive expression of Aβ1-42 and AβQ3-42 transgenic flies. (PDF 417 kb) [file 40478_2016_380_MOESM4_ESM.pdf]

Fig S5

**A**

**A $\beta$  1-42 LEVELS**

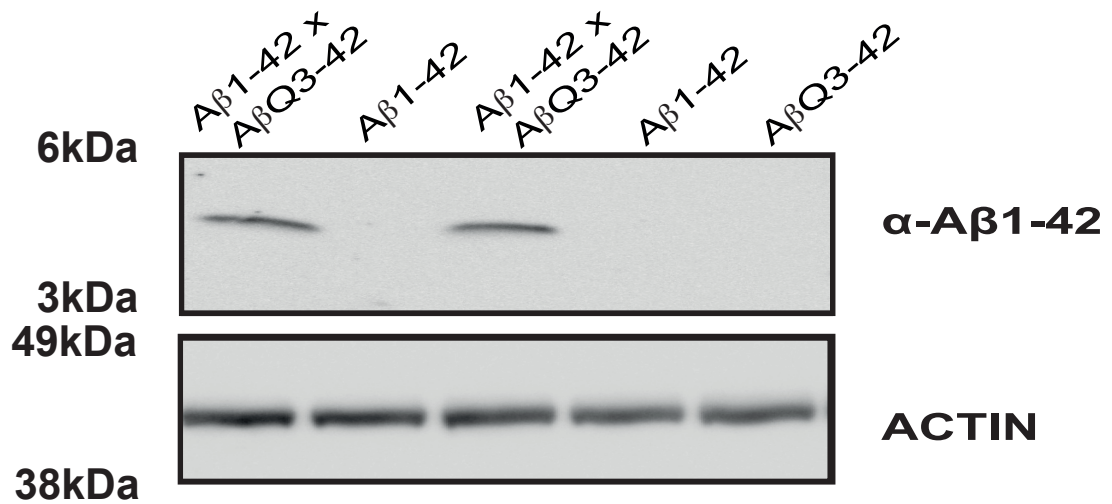

**B**

**LDS/SDS STABLE A $\beta$  1-42 Levels**

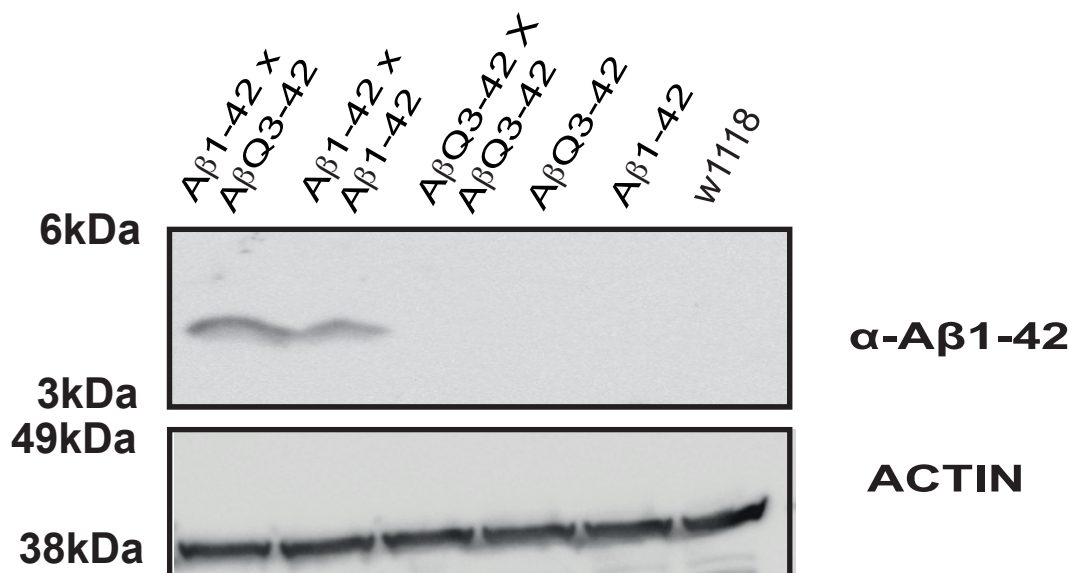

Supplement: Additional file 5: Figure S5. — pGluAβ increases accumulation of Aβ1-42 in vivo. (A). Flies co-expressing Aβ1-42 and AβpE3-42 peptide, had substantially more Aβ1-42 levels than flies expressing Aβ1-42 alone. Aβ was not detected in flies expressing a single copy of Aβ1-42 because it does not accumulate enough protein. However, Aβ1-42 was detected in flies expressing 2 copies of Aβ1-42 (B), confirming protein expression in these flies with the antibody. Furthermore, to validate specificity, Aβ1-42 was not detected in flies expressing either a single copy or double copy of AβpE3-42. GMR-GAL4 was used to drive expression of Aβ1-42 and AβQ3-42 transgenic flies. (PDF 10727 kb) [file 40478_2016_380_MOESM5_ESM.pdf]

Fig S6

A

EYE SCORING

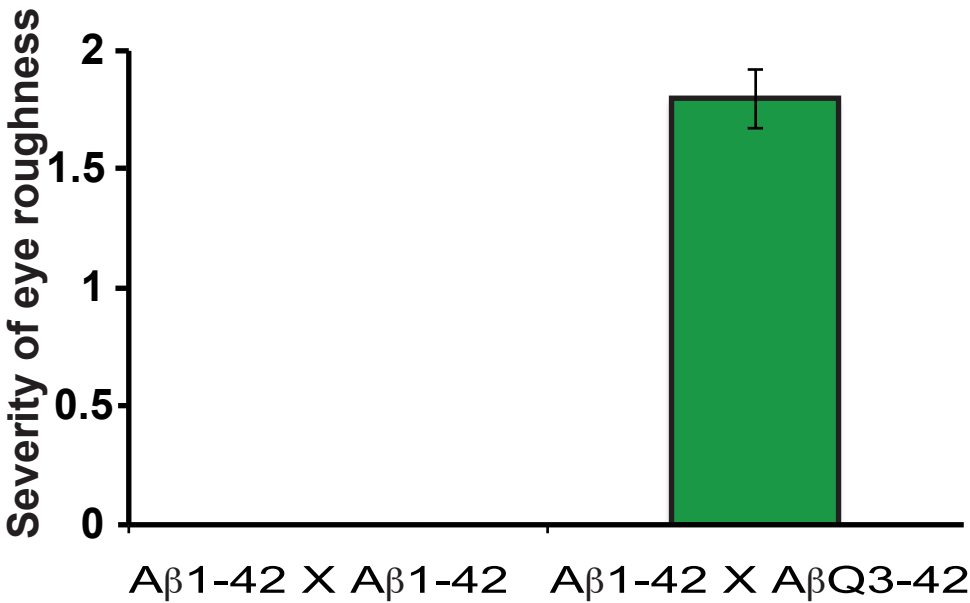

B

EYE SCORING

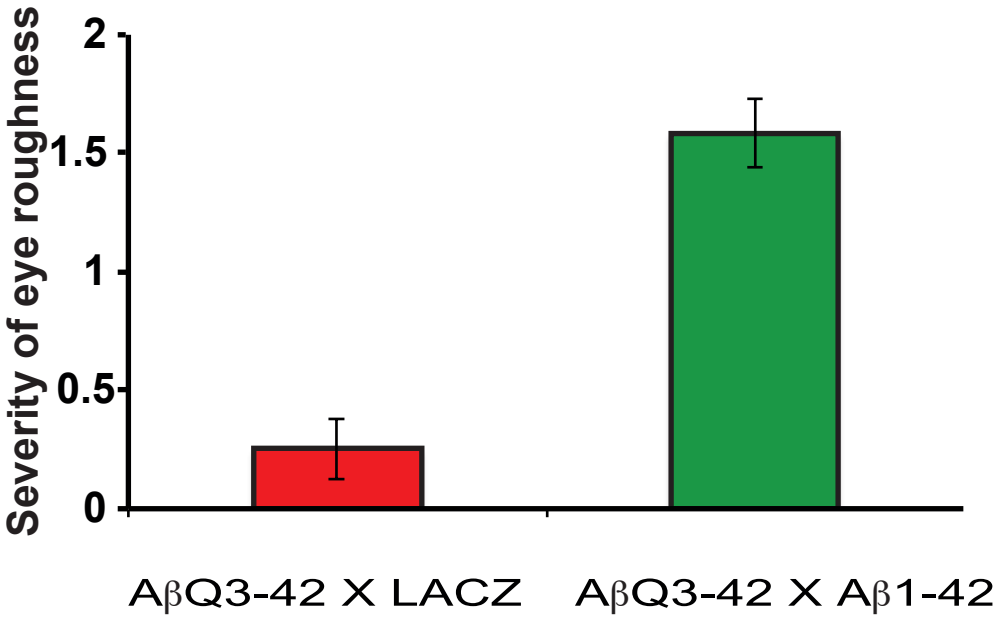

Supplement: Additional file 6: Figure S6. — Blind scoring of disorganised eye phenotype. The data demonstrate a significant difference in the degree of severity of eye roughness in flies co-expressing Aβ1-42 and AβpE3-42 in comparison to flies expressing either 2 copies of Aβ1-42 (A), or AβpE3-42 only (B), *P < 0.001 for both. Flies were grown at 25 °C. Data are presented as means ± SEM and were analysed by Student’s t test. GMR-GAL4 was used to drive expression of Aβ1-42 and AβQ3-42 transgenic flies. (PDF 303 kb) [file 40478_2016_380_MOESM6_ESM.pdf]
